# Palestinian and Norwegian Kindergarten Teachers' Perspectives on Psychosocial Support: A Qualitative Study

**DOI:** 10.3389/fpsyg.2021.761303

**Published:** 2021-10-26

**Authors:** Basel El-Khodary, Ingrid Christensen, Sanaa Abou-Dagga, Shawqi Raji, Susan Lyden

**Affiliations:** ^1^Department of Psychology, Islamic University Gaza, Gaza, Palestine; ^2^Department of Educational Science, University of South-Eastern Norway, Kongsberg, Norway; ^3^Department of Psychology, Hebron University, Hebron, Palestine

**Keywords:** psychosocial support, ECE, qualitative, mental health, education, comparative studies, kindergarten teachers

## Abstract

The current qualitative case study aims to explore and map the concepts and the conditions for providing psychosocial support in kindergarten across two vastly different countries, Palestine and Norway. The global challenge of providing psychosocial support toward children is increasingly acknowledged. Although education is described as crucial for psychosocial support from the health sector, studies dealing with the educational perspective on this topic are rare. Data from 26 participants (10 from Gaza, 10 from Hebron and 6 from Norway) were collected in qualitative semi-structured interviews. Despite vastly different contexts, the analysis showed some important common features. Kindergarten teachers in both countries link psychosocial support conceptually to psychological and emotional knowledge. The teachers in both countries are concerned about the psychosocial support being performed repeatedly in everyday situations, such as establishing routines, paying extra attention, and calming children and creating everyday safe spaces. They give detailed descriptions of the quality of their long-term, yet professional relations with the child. Time and space are crucial challenges in both countries, and they call for more knowledge on mental health. A main difference between the two countries was the role of the community and relation to parents. The Palestinian teachers defined psychosocial support as a “set of community services,” the teachers were frustrated with the lack of parental collaboration. The Norwegian teachers downscaled or overlooked the importance of community or parents and community. The findings give overall presentations of the concepts and the conditions for providing psychosocial support in education Palestine and Norway. We argue that education not only represents sites for conducting health-directed interventions but represents important resources for developing the field of psychosocial support in collaboration with community services. Education – and especially kindergarten provides other values, knowledge, and structural resources for the development programs and knowledge on psychosocial support.

## Introduction

Teachers' ability to provide psychosocial care for all children in kindergarten is critical. Mental health and well-being seem to be a common challenge across several countries around the globe, as about 20% of the world's children and adolescents have a mental health condition (WHO, 2021). The challenge for providing psychosocial support is widely acknowledged globally; for high- as well as low-income countries.

In recent years, there has been an increasing acknowledgment of education as a key factor for the promotion of development and health and the prevention of psychosocial and mental health problems (Adelman and Taylor, 2006; Soliman et al., 2020). The roles of the context and the relations in early childhood education make up the foundation for the developmental processes of young people and their mental health (UNESCO, 2016). The sense of connectedness, good communication, and perceptions of the care of an adult in schools are related to a wide range of mental health outcomes for young people (Soliman et al., 2020). These findings mirror global and universal challenges, where the school environment, education, and psychosocial support are essential contextual factors for the developmental processes of children and their mental health.

The current study takes a starting point in the field of psychosocial studies, a large and disparate domain of different approaches and disciplines. On a global level, the field of psychosocial support in early childhood is vast. Psychosocial support appears as part of psychosocial studies, developing interdisciplinary educational concepts across psychology, sociology, philosophy, and didactics. Mental Health and Psychosocial Support is a part of the general field of psychosocial studies, and is a composite term describing any type of local or outside support that aims to protect or promote psychosocial well-being and/or prevent or treat mental disorders (Inter-Agency Standing Committee, 2007).

### Benefits of Psychosocial Support in Child Development

The benefits of psychosocial support from early childhood and in education are widely agreed upon (UNESCO, 2016). Schools play significant roles in both developing the health or non-health of children (e.g., see the review of Ragnarsson et al., 2020). Schools are considered practical sites for professionals to reach each child with low-key and simple training programs (UNICEF, 2020).

However, the discursive use of the concepts of psychosocial support, its meanings, and practices are disparate. First of all, the combination of psychosocial support and education is conflicted, suggesting a separation of the enterprises of health and education. Many may associate psychosocial support with being a specialized psychiatric domain and providing treatment of trauma, anxiety, depression, etc. as diagnostic categories. According to this view, educators should not engage with the psychological issues of children, due to the risk of mistreatment and a lack of competence (Papadatou et al., 2002; Alisic, 2012).

Other approaches appraise the role of education in the interventions of healthcare and social care, often implemented by health agencies for educators to implement and follow. Despite acknowledging the significance of education for the health of children, the health sector is overrepresented in providing psychosocial support interventions. Educational approaches and interventions are requested (Haroz et al., 2020). There is a need for developing knowledge about the conditions and the concepts of psychosocial support from an *educational* perspective point.

Third, the global challenge of providing psychosocial support toward children is increasingly acknowledged, yet not as a contextual phenomenon. Thus, it can be fruitful to review the common features across different contexts, as well as highlighting some contextual factors for providing psychosocial support in kindergarten. Therefore, this study seeks to make an initial analysis to explore psychosocial support in kindergarten across two contrasting contexts, which are Palestine and Norway.

### The Contexts of Palestine and Norway

Palestine and Norway have very different conditions for providing psychosocial support in kindergarten. As a result of the long-lasting occupation of the West Bank and Gaza, Palestinians are exposed to violent conflict—especially those in Gaza, who have also endured a decade of blockade and closure—resulting in the fragile health and well-being of the population.

Previous study showed that individual and collective exposure to political violence has a negative effect on mental health (Giacaman et al., 2007). Up to 20% of the population of Gaza may have developed mental health conditions (UNICEF, 2015), and more than 300,000 children in Gaza may require some form of psychosocial care. A previous study showed that every child or adolescent has experienced at least one war-traumatic event (El-Khodary et al., 2020). Moreover, several studies have been conducted in the Gaza Strip showed that exposure to war-traumatic events leads to PTSD (e.g., Llosa et al., 2012; Kira et al., 2013) psychological maladjustment such as emotional disorders, lower self-esteem (e.g., Qouta et al., 2001), anxiety, and depression (e.g., Palosaari et al., 2013).

Norway is considered as a politically stable and peaceful country, having secure welfare and health system. Yet, both countries may deal with complex global issues of conflicts resulting in an increasing number of young people seeking help for mental health difficulties (Nordic Council of Ministers, 2017). The question about how kindergarten teachers describe psychosocial support toward traumatized children might be a key concern in both countries, as both manage the challenges of mental health, early intervention, and the particular role of kindergarten.

In Palestine, there is a high awareness of the psychosocial challenges of a country in conflict and the long-lasting exposure to traumatic events; thus, many strategies have been developed for health promotion programs, both through national instances and by non-governmental organizations (Al Ghalayini and Thabit, 2017; Joma et al., 2021). In Norway as well, there is an increased awareness of children's mental health (Holen and Waagene, 2014). Promoting children's mental health was first added to the framework plan for kindergarten in 2017, and for the last couple of years, the term “trauma-sensitive kindergarten” is under development. However, most of this knowledge exist as information to kindergarten and is more a result of political implementation rather than research-based knowledge (UNICEF, 2020). Despite the many differences, the two countries share the common challenge of developing psychosocial support as a concept and effective measures in kindergarten. This study, therefore, explores the kindergarten teachers' perspectives on the concept and the conditions for developing psychosocial support for kindergarten children in Palestine and Norway.

### The Term “Psychosocial Support” and Research on Psychosocial Support in Kindergarten

The term “psychosocial support” is widely used, although with several meanings. Psychosocial studies highlight psychosocial support as dynamic transactions between the environment and the inner psychological dimensions of a child (Woodward, 2015). Psychosocial support is then emphasized as being embedded in multilevel system interactions. Individuals, families, kindergartens, environment, municipality administration, society structures, and political situation influence and interact for the development of a child (Bronfenbrenner and Morris, 1998; Sabol and Pianta, 2012). The interaction between different societal actors affects a child's physical and mental well-being and their ability to function in society (Woodward, 2015). Another feature of psychosocial studies is a focus on the interactional quality and particular social patterns and transactions between the child and his/her caregivers.

Education is widely acknowledged as strategically important to reach out to children being exposed to traumatic experiences (Heltne et al., 2020). Taking an overall view of empirical studies of psychosocial support in kindergarten (and equivalent searches), Most empirical studies of psychosocial support take on the approaches and concepts from the health sector and its implementation into educational settings (Newland and Silver, 2020). Intervention programs seem to be an important role, such as training programs for trauma-exposed children in refugee camps, testing and promoting particular methods and techniques as “psychological first aid” program for teachers providing psychosocial support for children (e.g., Schultz et al., 2016). These intervention programs caught the attention of researchers in the field and were studied broadly in the Western and Arab world (Arafat and Boothby, 2003).

Many studies in education measure “psychosocial” as a dimension in a comprehensive and inclusive learning concept. The social and psychological dimensions have an impact on academic learning, reading, mathematical skills, and healthy development (Burchinal et al., 2016; Palmer et al., 2018; Froiland, 2020). Psychosocial support is also connected to the school setting in terms of improving behavior and preventing behavioral problems and dysfunctions of the child (Bierman et al., 2013; Mahon et al., 2020). Psychosocial support toward children with particular difficulties such as trauma, divorce, or child abuse is emphasized (Kolltveit et al., 2012; Bretherton et al., 2013; Bullock et al., 2019).

Some studies focus on the learning environment and the role of social and psychological support. Several studies focus on psychosocial support and consider psychosocial support as parental involvement in a child's life, either assessing the parental care or involvement of parents in providing psychosocial support (Zinsser et al., 2014; Shewark et al., 2018). However, the focus on psychosocial support and child development as a local and collaborative effort, is increasing. Heltne et al. (2020) provides an explorative study on how different local actors perceive of psychosocial support for children with adverse experiences.

However, although many studies elaborate on different aspects of psychosocial support, or describe and evaluate the use of programs for psychosocial support, few studies examines how the kindergarten teachers understand the concept of psychosocial support and the conditions for providing psychosocial support, in Palestine or elsewhere (Olsen, 2014).

### Research Problem and Questions

Studies taking a starting point in the educator's conceptions of psychosocial support may be rare to the best of our knowledge. The current study does not describe psychological dysfunction, diagnosis, or trauma treatment, but rather, aims to map the factors and dimensions that make up the conditions for providing psychosocial support according to kindergarten teachers. Kindergarten settings are not only sites for the healthcare interventions of psychosocial support; instead, this study shows how educators may bear their conceptual groundings and logic, promoting aspects of psychosocial support from an educational point of view. Thus, we attempted to develop the concept of psychosocial support as a practice through a grounded theory approach (Glaser and Strauss, 1967), exploring and mapping individual approaches to how psychosocial support is enacted in kindergarten and also producing a narrative of how issues of psychosocial support should be addressed (Woodward, 2015). We see psychosocial support as a broad term to be explored and mapped across two different contexts in Palestine and Norway.

The current study explores and maps the conditions for providing psychosocial support in education according to kindergarten teachers, in Palestine and Norway. It takes a grounded theory approach and is a case study in two regions of Palestine: The West Bank and the Gaza Strip. These findings are compared and contrasted with a sample study from South-Eastern part of Norway. In doing so, we aim to explore psychosocial support in kindergarten as a contextualized phenomenon.

The current study answers the following questions:

1. How do kindergarten teachers describe psychosocial support for children in Palestine and Norway?
2. How do kindergarten teachers describe their knowledge and skills in providing psychosocial support for children in kindergarten?
3. What are the kindergarten teachers' experienced challenges in providing psychosocial support?
4. What are the kindergarten teachers' professional needs in providing psychosocial support?

### Context Description of Kindergarten in Palestine and Norway

Palestine can be identified as a country that for a long time has been a “conflicted state” (Brandt et al., 2008), where children frequently have traumatic experiences, as opposed to Norway, a so-called “stable” and peaceful country (Olsen, 2014).

Kindergarten education in Palestine is defined as the stage where a child is between 3 and 5 years. Although kindergarten is not compulsory, the Law of Education (2017) in Palestine recommends at least 1 year of kindergarten,^1^ for all children at the age of 3–5 years. The policy of the Palestinian Ministry of Education (MoE) is directed toward integrating preschools into the formal education system. Additionally, a basic target of its current strategy (2017–2022) is to mainstream a preschool curriculum and increase the number of licensed kindergarten that meet health, safety, and professional standards. The Palestinian authorities emphasize the need for a child-friendly learning environment, where a child's personality can physically, mentally, and socially grow and get ready for basic schooling through games and other activities^2^.

The MoE's Statistics Manual (2019/2020) showed that there are 712 kindergartens in Gaza and 1,452 in West Bank, with 2,862 kindergarten teachers in Gaza and 4,548 in the West Bank (Ministry of Education, 2020). The number of children who benefit from educational and service opportunities in Gaza and the West Bank kindergartens is estimated to be 66,253 and 93,909, respectively.

Kindergartens in Norway are pedagogical institutions, providing early childhood education and care for children aged 0–5 years. Children start compulsory school the year they turn 6. The purpose and content are regulated by the Kindergarten Act (Ministry of Education Research, 2011), and all the kindergartens follow a national curriculum called The Framework Plan for the Content and Tasks of Kindergartens (Norwegian Directorate for Education Training, 2017). The latest figures for 2020 show that between 3 and 5 years, 97.3% of children are in kindergarten^3^, Kindergarten is subsidized by the government; it regulates the maximum a kindergarten place can cost every year.

Over 40% of the staff in the Norwegian kindergartens have a 3-year bachelor's degree in early childhood education and care, and other staff members usually have shorter vocational courses or experience. The ratio of staff to children is regulated by the government, although the level is seen as unsatisfactory to both parents and teachers' organizations, as the staff has to cover a long day—usually, kindergarten is open from 07:00–17:00—while working in shifts.

The core values of Norwegian kindergartens emphasize play as an intrinsic value of childhood. Kindergartens undertake a holistic approach toward children's development and work in partnership and agreement with their homes, to meet their needs for care and play; they promote learning and formative development as a basis for development. Preparation for academic work in school is a factor but is not majorly focused on in Norwegian kindergartens.

## Methods

### Study Design

This study is a comparative case study (Blömeke and Paine, 2008; Flyvbjerg, 2011), and takes on a qualitative grounded theory approach (Glaser and Strauss, 1967; Charmaz and Thornberg, 2020), to the psychosocial support of kindergarten teachers in Palestine and Norway.

The various cultural and contextual resources for developing practices, education, or programs on psychosocial support have different starting points. The study shares many of the challenges of conceptual validity of international comparative studies (Harkness et al., 2003; Ponterotto, 2006). However, the differences between the countries were seen as “extreme” cases for different approaches. Conducting a contrasting case study might show the variations and dimensions of the concept of the landscape of psychosocial support.

The data collection builds upon the qualitative answers to questions, resulting in definitions as well as narrative descriptions (Ponterotto, 2006). The analysis focuses on the kindergarten teachers' own descriptions and evaluations as a starting point for developing the concept of psychosocial support. The study has not, therefore, used pre-defined specified clinical indicators; instead, it poses open-ended questions regarding the teachers' concepts and conditions for providing psychosocial support.

### Sampling and Data Collection

In this study we aim at doing an introductory and explorative study. A randomized sampling of kindergartens has resulted in a total of 26 female respondents from the two countries, with 20 from Palestine (10 from Hebron and 10 from Gaza) and 6 from Norway. Some of the kindergarten teachers in Palestine hold a degree in early childhood education (ECE). However, all the Norwegian kindergarten teachers have a degree in ECE. The aim of this study has been to explore the conditions and conceptual basis for psychosocial support. Therefore, we first had a basis of Palestinian kindergarten teachers, and contrasting it with a smaller sample from Norway, for later elaboration.

Data were collected in September to October 2020 and selected through an open call by emails and direct contact with kindergartens. In Palestine, online interviews were conducted with the participants, in Norway, interviews were conducted either face-to-face or on Zoom (2 face-to-face and 4 on Zoom); the respondents replied through anonymized online forms.

Data were collected by native-language-speaking researchers through a qualitative semi-structured interview guide being developed collaboratively across Palestine and Norway, having both open-ended questions about how the teachers will define psychosocial support and describing the factors for providing psychosocial support along with its challenges.

### Interviews and Procedures

The interviews provide a thorough basis for exploring the particularity of individual experience and in-depth interviews of kindergarten teachers in Palestine and Norway. This approach is of interest in psychosocial studies. All the interviews were transcribed and then translated into English, being controlled by a second native speaker. The respondents have fictional names in addition to country abbreviations and numbers (H1, H2, H3, N1, N2, N3, G1, G2, G3, etc.).

### Analytical Strategies, Reliability, and Validity

The analytical strategy for the data incorporated a grounded theory approach (Glaser and Strauss, 1967), seeking to “bottom-up” the analytical categories, and from the teachers themselves. The study aims at seeing the concept *across* contexts and common themes for Palestine and Norway, and *in* context, showing some different trends between Palestine and Norway.

The grounded theory approach was applied in two steps: In the first step, we explored common themes across the contexts of Palestine and Norway. The results were presented as overall categories, following the strategy of so-called open-coding (Strauss and Corbin, 1990, p. 61). These categories were not predefined but stemmed from the data material. This first analysis led to what is labeled “Results part 1” with comparable categories in Table 1.

**Table 1:** Dimensions and emphases of psychosocial support in Palestine and Norway—Overall results of the study.

**Themes and Subthemes**	**Palestine**		**Norway**
	**West bank**	**Gaza**	
**Q1: Description of psychosocial support**			
Psychological/emotional support	30	8	10
Social and relational support	6	3	9
Cognitive and mental support	3	4	1
Behavioral support	3	1	1
Involvement of community apparatus	11	10	0
**Q2: Present knowledge and skills**			
Psychological skills and knowledge	7	10	10
Social and relational support	1	2	4
Particular method/skills/techniques	8	13	2
Experience/practice vs. formal	3	4	3
Moral/professional ethical skills and virtues	8	6	3
Involvement of community apparatus	8	9	3
Formal competence	3	2	9
**Q3 Challenges**			
Psychological and emotional challenges	4	7	4
Social and relational support challenges	1	4	4
Pedagogical challenges	2	7	6
Physical learning environment/time/space/resources	21	18	9
Community challenges (collaboration with instances and parents)	12	25	2
Formal competence challenges	14	0	0
Moral professional ethical challenges	0	4	3
**Q4 Professional development needs**			
Psychological and emotional knowledge	5	13	6
Social and relational support/skills	2	2	1
Pedagogical Methods and techniques	7	4	2
Practice and experience	7	7	0
Moral/professional ethical skills and virtues of learning and being	7	7	2
Involvement of community apparatus (parents, instances)	8	3	2
Formal competence	15	6	9

*Emphasized dimensions of psychosocial support in Palestine and Norway. The colours show the main emphasis of what the kindergarten teachers find important about psychosocial support. Red colour is large emphasis, orange is middle ranged emphasis and blank fields show little emphasis. The categories stem from the kindergarten teachers themselves*.

**Palestine:** Red colour and numbers 13–30 show major emphasis. Orange colour and number 7–12, show middle ranged emphasis. No collar and numbers 0–6 show little or no emphasis.

***Norway:** Red colour and numbers 9–10 show major emphasis. Orange colour and number 6-8, show middle ranged emphasis. No colour and numbers 0–5 show little or no emphasis*.

We did not conduct this categorization with any aim of establishing concept validity in a narrow sense (Elo et al., 2014); rather to give a brief overview over the different and some possible common features across the two countries. For the sake of conducting an international comparison, we chose to keep the coding lists as short as possible and aimed at revealing the main of each country. When we had agreed upon the main codes, we conducted a color code process. The color-coding process helped in establishing evidence for the reliability of data analysis throughout the whole material, which was repeated, discussed, and developed between the authors. Then the different codes were counted and put in a table to present an overview of the emphasis and tendencies between the two countries. The counting did not have any statistical means, as the number of informants was different between the data sets and the volume of the transcripts was also varying (e.g., shorter interviews in Hebron, longer in Norway). However, the numeric values of the coding indicate the number of meaningful units (phrases) making up thematic categories. The purpose of the thematic coding is to show the main themes as described by the kindergarten teachers and to provide an overview of perspective in the two countries (see Table 1).

The second step of grounded theory was “axial coding,” with results presented as short thematic narrations in the part called “Results, part 2.” This analysis links the concept of psychosocial support to contexts, to consequences, to patterns of interaction, and causes (Strauss and Corbin, 1990, p. 96). The analysis, therefore, has a second part accentuating the quotes and summarized the qualitative differences and some similarities when relevant between the Palestinian and the Norwegian kindergarten teachers. This second analysis shows the different perspective and approaches to the concept and the conditions of psychosocial support in kindergartens.

To meet reliability issues and agreement about the codes and the findings across the data sets, the analysis was conducted by three researchers (one from each sampling: Hebron, Gaza, and Norway). Then the categories and analyses were checked by the others and discussed, ensuring agreement about the analyses. According to Harkness et al. (2003), construct bias and method bias are the critical factors for achieving validity in a cross-national comparison. Cross-national comparisons remain complex because of the many triggers for bias (Ponterotto, 2006). The author team had close collaboration throughout the analysis in reading, suggesting, and discussing possible thematic codes that appeared in the material. This process also included the determination of thematic categories that were to be used for the comparison between the data sets.

## Results

In Palestine, ***in the Gaza Strip***, there were 10 kindergarten teachers, aged 28–45 years old, 7 have Bachelor degree and three have diploma, 4 have a degree in social studies while 6 have different specialties, their experiences in education at kindergartens range from 6 to 20 years. ***In Hebron***, there were 10 kindergarten teachers, aged 32–52 years old, 7 have Bachelor degree and one have diploma, 3 have finished high school education, their experiences in education at kindergartens range from 4 to 28 years. Kindergartens were both public and private.

In Norway, there were 6 teachers; one without any formal qualifications as kindergarten teacher, 3 with Bachelor in Early childhood, a kindergarten teacher, one Bachelor in child welfare and one Master in inclusive education.

In the following, we will present the results in two main sections labeled “Results part 1” and “Results part 2.”

### Results, Part 1: Dimensions and Emphases of Psychosocial Support in Palestine and Norway

Table 1 presents an overview of the different dimensions of kindergarten teachers' descriptions of psychosocial support in Palestine and Norway. It shows the main dimensions of psychosocial support as defined by the kindergarten teachers. It also shows a comparison of the emphases of psychosocial support in symbols. The symbol values of “major emphasis,” “middle ranged emphasis,” and “less emphasis” are adjusted according to the amount of data material in Palestine and Norway.

### Summary of the Research Questions in Part 1

Research question 1 (RQ1), which asks about descriptions of the concept of psychosocial support, shows that all the kindergarten teachers in Palestine and Norway linked psychosocial support to psychological and emotional knowledge. The Norwegian teachers described psychosocial support in terms of the relational quality between themselves and the children. Palestinian kindergarten teachers expressed a particular understanding of psychosocial support, stating it as connected more to the learning environment—like a community program or collaboration with the parents or other professionals. The community dimension was quite absent in the data from the Norwegian kindergarten teachers, who rather described the relational quality of teacher and child.

Research question 2 (RQ2) asks about the kindergarten teachers' description of their knowledge and skills in providing psychosocial support. The findings show that all the kindergarten teachers highly value their psychological knowledge and skills. The kindergarten teachers in Palestine underline that they employ psychological techniques, which only a few of the Norwegian teachers did. However, the Norwegian teachers underlined the importance of their formal competence.

When it comes to the main challenges that teachers face in providing psychosocial support research question 3 (RQ3), all the kindergarten teachers mentioned physical and material resources in the learning environment, such as money, enough space, and—at the heart of all the teachers—enough time for each child. Parental collaboration was a major concern for the Palestinian kindergarten teachers; they pinpointed it as the reason for psychosocial problems, along with a lack of common understanding of the kindergarten's role and some parents' lack of understanding of the role of care. Some Norwegian teachers were instead describing the challenges of methods. Although cooperation with parents was seen as integral, it was not mentioned as problematic.

Research question 4 (RQ4) shows that formal competence is requested in the West Bank and Norway, while psychological and emotional skills seem a prioritized need in Gaza. Despite the vastly different contexts, many kindergarten teachers in Palestine and Norway may have some major pedagogical challenges for developing the field of psychosocial support. The analysis resulted in several thematic overlaps, providing an overview of the conditions for providing psychosocial support in kindergarten. In the next section, we will present in-depth examples of the emphases of psychosocial support with the differences and similarities between Palestine and Norway.

### Results, Part 2: Qualitative and Narrative Comparisons of Psychosocial Support in Palestine and Norway

#### Research Question 1: How Do Kindergarten Teachers Describe Psychosocial Support for Children in Palestine and Norway?

In Palestine, kindergarten teachers define the concept of psychosocial support in different ways; some of them understand it as providing children who have faced stressful and traumatic events that affected their personalities with sufficient support and suitable environments, to help them to understand themselves and their abilities, be able to solve problems, achieve a considerable degree of psychological and social coping, and increase productivity. G1 (Afnan) says, “*My understanding of psychosocial support is a set of services that must be provided to the child when needed to get rid of the difficulties and pressures that s/he faces.”* Similarly, many kindergarten teachers explain psychosocial support as services that are provided to the children and their families to achieve psychosocial coping. The teachers accentuate that psychosocial support should include the child and his/her family, especially those children who experienced stressful life events such as domestic violence. G4 (Faten) explains psychosocial support as, “*…services which are provided to children and their families which include prevention and treatment programs that make children and their families compatible psychologically and socially and increase their productivity, awareness, and motivation in all areas.”* H5 (Amani) says that psychosocial support is to, “…*provide psychological support to the child and the family in issues that the child is struggling with including domestic abuse, aggression, etc.”*

A third group defines “psychological support” as providing children with the social skills that enable them to fulfill their social duties by the prevailing values and standards in society. G10 (Wafaa) says, “*The psychosocial support from my viewpoint refers to improving children's and their families' psychological and social health, by increasing social interaction among them and improving the relationships among the children themselves.”* Another group of teachers gives more attention to engaging kids in healthy relationships and providing chances to express opinions and thoughts; they try to understand the behaviors from the child's perspective and avoid using punishment. H7 (Roba) refers to psychosocial support as, “*…encouraging the child to talk and listen, avoid using threats and punishments, and sharing everything about the child with his family.”*

In Norway, psychosocial support as a term seems to be less known. Some kindergarten teachers seemed a bit surprised by it. N2 (Kari) says, “*It's not a word we use in kindergarten, “psychosocial support?”* N5 (Ida) says, “*Well, I am not used to using that concept but I can describe it as being there and helping a child to find ways to behave towards other children.”* In the Norwegian dataset, however, many of the teachers describe the importance of psychological and emotional support; psychosocial support is connected to the personal space between the teacher and the child. The terms “feelings” and “emotions” are repeated. For instance, N1 (Anna) says, “*It is about meeting children's emotional needs. That's what I think of first”* while N6 (Brit) says, “*To be available for the children and their feelings, and even to mirror the feelings.”* The kind of feelings is not described in detail. N1 (Anna) expresses psychosocial support as, “*…a need for regulation of feelings, support in dealing with difficult and strong feelings.”* Some of the teachers describe feelings in terms of a “space” and “meeting the children where they are” (Brit, N6). This leaves an impression that psychosocial support is a kind of here-and-now space and personal relation with the child.

#### Research Question 2: How Do Kindergarten Teachers Describe Their Knowledge and Skills in Providing Psychosocial Support for the Children in the Kindergarten?

When the kindergarten teachers describe their knowledge and skills in providing psychosocial support, one factor is important: Experience. Kindergarten teachers in Palestine clearly state that they have professional experience, but they still need more knowledge and more training in psychosocial support and kindergarten teaching in general. H10 (Abla) says, “*I have knowledge and experience in kindergarten psychosocial support approach but still need more experience and more training.”*

Furthermore, Palestinian kindergarten teachers believe that college provides them with the academic knowledge but not the practical psychological skills that they need to be a teacher who can offer psychosocial support. Teachers rely more on their own experience, which was built during working years, than on professional experience. G5 (Fatimah) says, “*Honestly, from my experience in dealing with children, I may have some knowledge and skills but not like specialists in this field. I have been trying to find solutions. I don't deny that experience plays a main role. We learn by trial and error.”* H9 (Nila) says, “*My experiences and skills are limited and simple. I usually deal with my kids as a mother and not as a psychosocial supporter. I very much rely on my skills as a mother than a psychosocial supporter.”*

Kindergarten teachers believe that they have inadequacies in terms of ECE knowledge and skills, and they always need supervision, consultation, and follow-ups from a specialist, such as a school counselor or a mental health specialist. H8 (Hania) says, “*I have the knowledge and skills when I am within my capacity and once the issue is above my capacity, I refer the child to a professional counselor.”*

All the Norwegian teachers emphasize also experience as a source of professional learning. Many teachers claim high formal competence due to their bachelor's and masters' degrees, in addition to formal courses in psychosocial support; however, they seem to pose questions about the value of formal competence in meeting the difficulties of a child. Some of the Norwegian teachers also refer to the “Circle of Security” as a particular theory and methodological approach for attachment and psychosocial support (Woodhouse et al., 2018).

One of the Norwegian teachers divides between “regular problems” (N4, Kari) that she feels she can deal with, as opposed to “difficult cases and mental issues,” stating that “…when a child really is showing that they are struggling, it is quite painful to feel you are not up to the challenge.” They need external support from specialists to cope with some situations.

All the kindergarten teachers express an urgent need for both formal competence and training in ECE to provide psychosocial support. They describe the need for expertise and supervision. In both countries, there is confusion regarding the status of psychosocial support. On the one hand, it is considered as a “specialist” domain, to be provided outside the kindergarten. Another finding from the interviews is that the kindergarten teachers seem to distance themselves from being what they call “specialists,” implying: There is a gap between the “regular” they deal with in the kindergartens and the issues that belong to other sectors in their everyday lives. For some teachers this appears as a clear inferior position. On the other hand, the teachers also define themselves as important providers of psychosocial care. The Palestinian teachers tend to be more confident with their competence than their Norwegian counterparts.

#### Research Question 3: What Are the Challenges Experienced by the Kindergarten Teachers in Providing Psychosocial Support?

Kindergarten teachers reported several challenges that they face when providing psychosocial support to children. First, teachers in Palestine and teachers in Norway are on the same page regarding the staff-to-child ratio, stating it as one of the most difficult challenges they face when providing support to the children; these can be seen as *materialistic challenges*. In Palestine, the staff-to-child ratio was often mentioned together with the small sizes of the rooms, like H1 (Hiam) states: “*Firstly, in the kindergarten where I work, there is a huge number of children. Huge numbers hinder us from providing children with sufficient psychological support.”* H1 (Hiam) says, “*The number of kids in one classroom is too much and does not give me enough time to follow up on the needs of every child.”* There is also a lack of toys and other resources. G6 (Kafa) says, “*The lack of resources is a challenge. I have 25 children in the classroom. Children can't all play at the same time due to the lack of sufficient toys. Therefore, I ask some children to play and the others to wait.”* G9 (Hiam) says, “*A challenge I face is the small size of the classroom. I can't practice most of the activities and not all children can play with me, especially when I want to use educational methods.”*

Similarly, in Norway, kindergarten teachers reported the staff-to-child ratio as one of the major challenges that they face when providing psychosocial support. N3 (Peter) says, “*The first I think of are the numbers, the number of children and the number of staff.”* N2 (Kari) stated, “*…that our special education provision is just not enough.”* Elaborating on this, the teacher says, “*One frustration is that all the children mentioned have been assessed by the PPT, but they have not been given enough hours of extra help.”* PPT is the local pedagogical-psychological service overlooking kindergartens and schools in the municipality, but only sometimes their assessments of the children lead to the employment of more staff.

Teachers in both countries mentioned that the overall demands of leading a kindergarten group are a sort of an administrational challenge connected to curriculum achievement. G1 (Afnan) says, “*The first challenge is the great responsibility we should as pre-school educators, which includes the tasks we carry out inside the kindergarten, such as report writing, activities, and events.”* It is mentioned that time is a common factor, especially the time for planning, evaluating, and documenting one's teaching. G10 (Wafaa) says, “*I don't have enough time to support children. There's a curriculum that I should finish teaching in a limited time. I should stick to the curriculum plan I have. I don't have time to assign outdoor activities to the children or support them psychologically.”* H3 (Manal) says, “*I don't have a teaching assistant in the classroom, so I don't even have a day off, and I cannot leave the kids with anyone in case of my absence.”* Similarly, the pressure of time seems to be a challenge for kindergarten teachers in Norway as well. N6 (Vera) says, “*There are so many things we have to do—we have to plan, and write reports, do this, do that.”*

One significant difference between Palestine and Norway is the kindergarten teachers' relationship with the local community, especially with the parents *(social challenges)*. The teachers in Palestine report a lack of family cooperation and cases of family denial when informed of their child's problems. G8 (Heba) says, “*One challenge is that parents may be unresponsive to their children's problems. Some parents don't believe that their child does anything.”*, meaning that they underscore any mistakes by their child. G5 (Fatema) states, “*Parents' carelessness and devaluation of any problem are a big challenge for us in providing psychosocial support.”* Likewise, the kindergartens in Hebron encounter the same challenge. H1 (Hyam) says that there is a “*lack of cooperation from the family side…*[and that the family] *does not share the information with the teacher.”* They also seem to describe the relationship and the expectations from the parents as a key tension. H3 (Manal) says, “*The lack of cooperation and the lack of understanding from the families make my job harder*. Financial status is also one of the challenges that are faced by kindergarten teachers. Some families are poor and that negatively affects cooperation with the kindergarten children. G8 (Heba) says, “*Another challenge is the financial status of some children. Some families are poor. In this case, I should get in touch with well-off families to find a solution for this problem and ask them to donate an amount of money.”*

The findings from Norway also mention the parent collaboration, the need to cooperate, exchange information, and provide support to parents. However, none of these issues are mentioned when asked for the prevailing challenges.

Interestingly, lack of knowledge was also seen as one of the challenges in providing adequate psychosocial support. H10 (Abla) says, “*I have knowledge and experience in kindergarten psychosocial support approach but still need more experiences and more training.”*

N5 (Ida) says, “*It is important to have staff who understand the importance of this* [supporting the children's feelings], *who can understand and know how to get involved with the children.”*

The first challenge in providing psychosocial support for the Norwegian teachers is giving the child enough space to develop on its terms. Anna (N2) says, “*There is a need to work on this…on how the children can come more to the fore in kindergarten, where all from their wishes, needs and ways of being are given more space.”* The Norwegian teachers' language about the child's needs, to pay attention to the child's “space” and to respect the “voice” of the children may mirror the United Nations Convention of the Rights of the Child, §12.^4^ Respect for children's views has been a central feature of the Kindergarten Act for a couple of decades (Ministry of Education Research, 2011). Psychosocial support may be seen as a relational quality, in that the staff follows the child rather than the child following the teacher. A change of the pedagogical mandate with the “adult world” can diminish the agency of the child. As N1 (Peter) states, “*So, the challenge is to include the child in good interactions, good play without hurrying the child*.”

A second challenge can be described as a change of the pedagogical mandate of the kindergarten from free play into a school-like productivity rationale. Anna (N2) describes a kind of pressure from adults as well: “*Children are pressured— ‘pressured' into a form dictated by adults and where children have less opportunity to contribute, where children's participation is marginal and where the adults' needs are more visible. They can take up too much space*.” The “pressure” and the “hurrying” by the staff might come from the increased focus on acquiring school knowledge, such as mathematics, science, and the Norwegian language.

Third, there is also an administrational challenge of increased documentation and productivity demands in the kindergarten. The informants mention “many routine situations” and the time used in documenting and writing reports, giving staff less time to follow up interests and issues initiated by the children.

#### Research Question 4: What Are the Kindergarten Teachers' Professional Needs for Providing Psychosocial Support?

In Palestine, kindergarten teachers expressed their need for more training and more educational updates for their information and skills in the ECE field, to be confident in handling the kids' issues and successful in providing actual psychosocial support services. G6 (Kafa) says, “*I seek to improve my abilities. Children are an encyclopedia; one feels worthless before them…I would like to improve my abilities in dealing with children. I would like to do an MA in dealing with children to know how to deal with them. We are ready to learn and do anything.”*

In Norway, kindergarten teachers expressed more concerns about the child's feelings and having the proper education and proper skills to be up to the challenges of being a psychosocial support teacher. N2 (Kari) says, “*…when a child really is showing that they are struggling, it is quite painful to feel you are not up to the challenge.”* N3 (Peter) announces the need for, “*knowledge on how a trauma can affect a child's psychological development and how I can support [the child].”*

Kindergarten teachers stated that they need more education and knowledge, training, and skills to be able to provide good psychosocial support to children. When describing the first time she welcomed newly arrived refugee children with war trauma to kindergarten, N1 (Anna) says, “*It wasn't easy to make a plan and I didn't feel I had a lot in my pedagogical bag of knowledge, in a way.”* N2 (Kari) mentions that the kindergarten teachers have conferred with the childcare services. She accentuates the need for all staff working directly with the children to have some level of basic pedagogical knowledge: “*In a way, they have competencies—practical experience after many years working in kindergarten—but no basic education course or pedagogical knowledge as a foundation. So, without that, working with children can be unfortunate, especially for children that need a little more help.”*

The Norwegian teachers emphasized child growth and brain development as areas that all teachers need to be aware of and trained for. N3 (Peter) says, “*In this kindergarten, we have, in these last few years, spent a lot of time learning about brain development.”* Teachers mentioned the possible access to available resources and lack of cooperation with outside-school resources. N4 (Vera) says, “*No, no, we don't get much input from outside. We have to have confidence in each other and discuss together.”*

About half of the teachers showed interest in each of the following training topics: brain and psychological development, children, and trauma and how to handle trauma and crises, cooperation with parents, conversation skills and how to ask questions, how to communicate with families/parents, and learning English to communicate with English-speaking parents.

## Summary

### Summary of the Results in the Study

In this study, we have mapped and explored the kindergarten teachers' perspectives on the concept and the conditions for developing psychosocial support for kindergarten children in Palestine and Norway. The present study represents an initial exploration of an educational approach to the field of psychosocial support. Followingly, this study has taken on a grounded theory approach that both seeks common features and differences in kindergarten teachers' descriptions of psychosocial support in Palestine and Norway. In the study, we asked how the kindergarten teachers in Palestine and Norway conceptualize psychosocial support (RQ1), about their knowledge and skills (RQ2), about the major challenges for providing psychosocial support (RQ3) as well as their further needs (RQ4). The study has resulted in an overview of the kindergarten teachers' concepts and gives insights into their emphases and conditions for providing psychosocial support in Palestine and Norway.

The analysis brings forth a varied concept of psychosocial support, yet with many similarities between the two countries. Psychosocial support can be summarized as five key dimensions, such as (a) psychological/emotional support, (b) social/relational support, (c) cognitive/mental support, (d) behavioral support, and (e) Involvement of community apparatus.

The analysis of the first research question (RQ1) showed that the concept of psychosocial support is more well-known in Palestine than in Norway. The kindergarten teachers all emphasize the work with getting to know children well and define psychosocial support as primarily an emotional, individual domain, caring, observing, and engaging with the child. The Palestinian teachers request and depend more on the community services and parents while the Norwegian teachers gave in-depth descriptions on the personal relations and promoting the agency of the individual child.

Research question 2 (RQ2) asked about the skills for providing psychosocial support. The kindergarten teachers in both countries emphasize and take pride in their experience, spending time, and “mothering” as the main qualifiers of providing psychosocial support. They acknowledge and call for the support of the health sector and more specialized knowledge, seeing themselves as inferior.

The results from research question 3 (RQ3) about the main challenges for psychosocial support showed that the Palestinian teachers were the collaboration with the parents, as well as an urge for specialists to supervise teachers. The Norwegian teachers were less engaged about the parents but reflected critically about their pedagogical role and their personal relation with the child, describing the issues of establishing safety and trust with the child, as well as promoting the child's own voice and agency.

The result from research question 4 (RQ4) about the kindergarten teachers' professional needs, shows that all the kindergarten teachers express an urgent need for both formal ECE competence and training for providing psychosocial support. They both describe the need for expertise and supervision.

## Discussion

To our knowledge, this is the first study to highlight psychosocial support from kindergarten teachers' perspectives in two different countries. In conducting a contrasting and comparative case analysis between kindergarten teachers in the two countries Palestine and Norway, we acknowledge and expect widely different situational, historical, and personal contexts for being able to conceptualize psychosocial support. The current events mirror Palestine as a country in constant conflict and war, while Norway represents a country of long-lasting peace. Yet, conducting this comparison is of high importance, both as a global common challenge on the one hand, and a contextual phenomenon on the other. The findings are of importance for the further development of education and training in kindergartens, as well as in community development programs for psychosocial support to children and families, being at the heart of worldwide attention and strategies (WHO, 2019).

### A Methodological Note: Similarities Across Widely Different Contexts

In this study, we expected large variations in the conceptualizations and the practices of psychosocial support between the two countries. One possible critique could be raised to the procedures of conceptual validity, which undoubtedly has been a challenge in comparing findings from different countries such as Palestine and Norway. In this study, this gap is made as an introductory and explorative study to map variations in the concept and conditions for providing psychosocial support in kindergarten, with a small number of cases. The findings suggest a conceptual variation to be explored further. However, we were also surprised by how many *similar themes* and emphases of kindergarten teachers identified in the data material from Palestine and Norway. One explanation of the similar findings could be the global focus on the need for psychosocial support in education from international agencies (e.g., WHO, 2019; UNICEF, 2020).

Dealing with the development in early childhood and the acknowledgment of the vulnerability of children in conflict and instability is a global concern in both high- and low-income countries (WHO, 2019), such as Norway and Palestine. Furthermore, international agencies, such as WHO, UNESCO, UNICEF, and other global agencies have developed numerous programs of psychosocial support, and for low-income countries and countries in emergencies, establishing psychosocial support as a field the two last decades (Beaglehole et al., 2018). Thus, a unified discourse on psychosocial health and psychosocial support might have become mainstreamed, also resulting in the similarities found in the study.

### The Need for and the Possibility of an Educational “Footprint” in Providing Psychosocial Support

This article might envision some possibilities and resources that kindergarten and education could provide. Among many relevant discussions, we will raise an important and foundational question: Why do we need a particular pedagogical “footprint” in the conceptualization and the practice development of psychosocial support in kindergartens?

The development of the field of psychosocial support is urgent, and during the next years, the interventions and services across community-based, general health will be one important focus (WHO, 2019, p. 2). However, this present study shows that community sectors, such as health, are described as important, yet distant by the kindergarten teachers. As research shows that the health sector is over-represented in interventions of psychosocial support, there is a need to integrate psychosocial support in other sectors, such as education (Haroz et al., 2020). Psychosocial support has, to a large degree, been developed by the domains of psychology and psychiatry. Education is envisioned as a profitable and effective space for providing psychosocial support (Haroz et al., 2020).

What is the potential of education providing psychosocial support? We will argue that education and kindergartens are not only convenient *sites* for conducting health-initiated programs in terms of being time- or cost-effective compared to e.g., clinical treatment or limited interventions. Despite the indisputable need for mental first aid in conflict areas, education might do more than for instance conducting a program calming children with post-traumatic reactions, or use different methods for treatment involving conversation techniques, role play, drawing, etc. (as described by e.g., UNICEF, 2020). Instead, we can identify three insights from the study that education may provide to the concept of psychosocial support:

First, the study gives important insights about psychosocial support as a particular educational *value-oriented domain*. At the heart of psychosocial studies is the focus on the interactional quality and particular social patterns and transactions between the child and its caregivers (Woodward, 2015). Where a therapist can be dependent on a momentary “alliance” (Stubbe, 2018), many of the kindergarten teachers in this study are engaged in *being available* to a child. The teachers describe the challenge of having time to recognize the actual and exact need (Rq3); whether it is to adjust the morning routines or giving the child a hug, using the child's natural play situations, and being open to bodily expression. This implies more to a professional relationship than a therapeutic one; the challenge for the teacher is to develop care and love as professional domains (Arendt, 1998/1958, p. 181). However, a foundational problem raised by the kindergarten teachers, it the constant challenges of not having time and not having space to act properly on their professional values.

This present study reveals how kindergarten teachers represent a professional resource for *professional and holistic knowledge of* children. The teachers all describe how they see psychosocial support as measures for the child's emotional, physical, and cognitive development, highlighting a resilience perspective, seeing good mental health and developmental outcomes, despite exposure to significant adversity (Tol et al., 2013). Having a broad focus on local conceptualizations of psychosocial support is an overall and critical factor for the success of interventions.

Finally, we will accentuate the potential role that kindergartens can play as professional coordinators and collaborators in community interventions. In this study, and from RQ2 and 4, we see that the teachers feel skilled, ready, and motivated to engage and provide psychosocial support. In Palestine, the teachers are even more concerned with contributing as local actors and collaborate with parents and community services. Although the Norwegian teachers emphasized the community as less important, the cross-sectoral approach is vital for the effect of school-based programs for mental health (Gjerustad et al., 2019). Reviews of psychosocial support interventions need to be realized through coordinated and complementary actions within the multiple sectors and clusters of the humanitarian response (Ran, 2019, p. 18). Again, the Bronfenbrenner and Morris (1998) ecological model challenges communities to collaborate across sectors close to the child, as is also accentuated by Heltne et al. (2020) As the infrastructure of a public kindergarten seem to develop in Palestine as it has done in Norway, kindergartens might represent a key actor for connecting the child, parents, and community services.

This present study has explored the concept of psychosocial support, with a bottom-up approach to what may be important in kindergarten in two different countries. This might serve as an initial investigation to be taken further by new studies, for instance systematizing a holistic perspective on psychosocial support, and from different actors in a community. As one size does far from fitting all, there might be a need to develop psychosocial support as contextually encompassed and educational domains (Wessels, 2017). In that respect, it is of great importance to developing studies that critically examine more deeply the contextual basis for psychosocial support, revealing the conceptual and practical groundings for the field of psychosocial support.

## Limitations and Implications for Future Research

The current study provided qualitative evidence of the construct of providing psychosocial support on a phenomenological level. However, the current findings should be interpreted with caution due to some limitations. *Firstly*, the findings were obtained by conducting qualitative semi-structured interviews and allocating teachers' responses to categories derived by the authors from the psychosocial support interview guide developed collaboratively across Palestine and Norway. Even though the authors took caution to be objective and transparent, categorization of textual material remains a subjective procedure due to the interpretative paradigm of qualitative research. *Secondly*, the results obtained by the personal interviews which may have subjective bias when answering the questions. *Finally*, the number of the sample was small (26 participants) and only 6 from Norway. Therefore, the obtained results may be generalized across a larger kindergarten teachers' population.

Although of these limitations, the findings of the current study may be interesting for parents, kindergarten teachers and ECE educators regarding how to raise awareness of the importance of providing psychosocial support for children and the necessity of collaboration of parents with kindergarten teachers. Furthermore, the findings may be interesting for governmental bodies and policy makers in terms of the polices which should be followed regarding number of children in each class, enough places for play, allocating teacher assistance as well as providing the kindergarten with sufficient materials resources such as games.

For future research, this study may be seen as a provider of qualitative foundations of relevant key issues for approaching the field of psychosocial support in kindergartens, and for future development of indicators.

## Data Availability Statement

The raw data supporting the conclusions of this article will be made available by the authors, without undue reservation.

## Ethics Statement

The studies involving human participants were reviewed and approved by Norwegian Centre for Research Data. The patients/participants provided their written informed consent to participate in this study.

## Author Contributions

BE-K coordinated the manuscript. BE-K and IC drafted the overall manuscript. SL, SR, and SA-D collected data and conducted analyses. All authors conceived the study and its design.

## Funding

This study was funded by the project NORPART-2018/10166, Developing the Teacher Education in Pedagogy for Early Childhood Education and Early Elementary School in Palestine and Norway.

## Conflict of Interest

The authors declare that the research was conducted in the absence of any commercial or financial relationships that could be construed as a potential conflict of interest.

## Publisher's Note

All claims expressed in this article are solely those of the authors and do not necessarily represent those of their affiliated organizations, or those of the publisher, the editors and the reviewers. Any product that may be evaluated in this article, or claim that may be made by its manufacturer, is not guaranteed or endorsed by the publisher.

## Footnotes

^1^ http://www.moehe.gov.ps/moehe/ministerialsystemsandregulations

^2^ https://andp.unescwa.org/sites/default/files/2021-07/palestine_education_sector_strategic_plan_2017-2022.pdf

^3^https://www.ssb.no/en/utdanning/barnehager/statistikk/barnehager (accessed July 4, 2021).

^4^https://www.ohchr.org/en/professionalinterest/pages/crc.aspx (accessed July 7, 2021).
